# Supplementary material for: Are carbon emissions trading and green financial instruments synergistic? -Comprehensive quantitative research based on content analysis
Source: PLoS One. 2024 Mar 7;19(3):e0298601. doi: 10.1371/journal.pone.0298601 (PMC10919601; doi:10.1371/journal.pone.0298601)
Supplement: S1 Data — (ZIP) [file pone.0298601.s001.zip › Data/地级市Z_F工作报告.zip!/╔┬╬≈/║║╓╨╩╨/└┤╫╘╣1⁄2╓┌║┼í╛┬φ┐╦╩2╛▌═°í┐_2019.docx]

各位代表：

　　现在，我代表市人民政府向大会作工作报告，请予审议，并请市政协委员和其他列席人员提出意见。

　　一、2018年工作回顾

　　过去一年，全市上下以习近平新时代中国特色社会主义思想和党的十九大精神为引领，在省委、省政府和市委的领导下，紧扣新时代追赶超越、高质量建设“三市”目标，以开放倒逼改革、以改革激发创新活力，统筹推进稳增长、促改革、调结构、惠民生、防风险各项工作，经济社会发展呈现稳中有进、进中向好、质效双增的良好态势，圆满完成了市五届人大三次会议确定的各项目标任务。

　　——主要指标稳增。实现生产总值1471.88亿元，增长9.5%、增速居全省第2位、连续七个季度稳居全省前三。规上工业增加值494.01亿元，增长14.5%、增速居全省第3位。全社会固定资产投资增长14%、增速居全省第3位。社会消费品零售总额469.3亿元，增长11.5%、增速居全省第3位；外贸进出口总额突破10亿元大关，达到10.9亿元。

　　——发展质量向好。地方财政收入49.96亿元、增长9.5%，其中税收收入占比74.8%、高于省上下达任务4.8个百分点。规上工业利润增长15.2%，企业效益持续好转。军民融合产业产值增长20%，高于规上工业总产值增速3个百分点。战略性新兴产业增加值增长12.5%，高新技术产业产值增长18.7%，创新驱动能力不断增强。新培育“四上”企业436户，市场主体达19.24万户、居全省第4位。新增陕西名牌18个，质量工作获全省优秀等次。

　　——县域经济共进。6个县区生产总值超过百亿元，汉台区生产总值突破300亿元。在全省县域经济监测考评中，城固、南郑跻身“陕西省县域经济社会发展十强县”，勉县上升13个位次，洋县和留坝均上升10个位次，镇巴上升8个位次。

　　——民生事业扎实。全年民生类支出280.3亿元，占财政总支出的82%。城乡常住居民人均可支配收入分别达30380元、10088元，增长8.5%、9.3%，增速居全省第1和第3位。全市贫困发生率由2015年底的17.96%下降到5.15%。全面完成234所义务教育学校改薄工程。医疗医保联动改革得到中省肯定。五项社会保险参保率均达98%以上。中心城区空气质量优良天数294天，同比增加25天、增幅居全省第一；全域水质保持优良，水环境质量居全省前三。

　　——对外形象更靓。以高铁、高速、机场为骨架的立体交通网络畅联全国，汉中海关获批设立；全国首届健联体高峰论坛、贫困人口慢病签约服务现场推进会在汉中召开；“汉风秋月”中秋晚会、“天汉传奇”实景演出广受好评，天汉湿地公园醉美芦花成为“网红打卡地”；话剧《四叶草》、微电影《守护》等“汉中故事”传扬全国；国际留学生感知汉文化活动联通中外；首届朱鹮国际论坛、全球创新创业教育论坛暨产教城融合发展论坛、全球旅游推介暨世界旅游小姐总决赛成功举办，汉中进入“高光时刻”。

　　一年来，我们主要抓了以下六个方面的工作：

　　（一）壮大支柱强引领，三次产业协调并进。通过优化布局明晰发展路径，创新机制强化园区承载，完善政策聚集资源要素，招大引强培育骨干企业，推动“六大产业”加快发展，三次产业结构由上年的15.8：46.3：37.9调整为14：47.7：38.3。工业经济提速增效，规上工业总产值1580.08亿元、增长17%；装备制造、现代材料、高品质食药三大支柱产业产值1359.61亿元、增长17.4%；21个重点工业园区营业收入1163亿元、增长15%；工业投资增长40.2%，同比提高35.4个百分点；非公经济增加值占比53%，同比提高0.9个百分点。现代农业稳步推进，农业总产值363.23亿元、增长3.7%；粮油生产保持平稳，十大绿色农产品基地持续壮大；60余家农业企业入驻京东云仓，市外开设农产品销售门店350余家；新增“三品一标”农产品80个，国家地理标志产品达20个、居全省第1位，山茱萸、杜仲叶、天麻、西洋参被列入国家药食同源目录；洋县成为全国有机产品十佳县，“城固猕猴桃”率先认证出口欧盟；振兴乡村产业服务公司组建运行。旅游三产活力显现，天汉文化公园、汉山景区等26个市级文旅项目完成投资92.5亿元，兴汉新城文化旅游核心区局部展示，留坝栈道水世界建成4A景区，培育“汉家乐”27家，全年旅游总收入306亿元、增长32%；宁强被评为全国首批国民休闲旅游目的地和陕西省全域旅游十强县。京东云仓及城市仓建成运营，10个贫困县区创建为全国电子商务进农村综合示范县，村级电商站点实现全覆盖，电子商务交易额列全省第3位。

　　（二）推拉结合抓项目，发展支撑更加有力。深入开展项目建设主题年活动，以项目动态管理为抓手，坚持半年一考核一调整，加大对投资增速、进入市级盘子项目和产业项目、重大项目的考核权重，着力形成“九牛爬坡、个个出力”的倒逼机制，项目建设加快推进，472个市级重点项目完成投资931亿元，占年计划的109%；全年引资到位735亿元、增长18.5%，实际利用外资3067万美元。产业项目方面，航空零组件制造、邦华新能源电动汽车电源总成、汽车电控节能变速箱生产线、陕钢废钢回收加工等项目竣工投产，陕飞扩能配套、天谷药业中药材加工及物流、丰泰纺织工业园等项目有力推进，汉钢集团短流程炼钢、汉王药业迁扩建等项目开工建设。交通项目方面，汉中至巴中高速通车，秦巴深山环线的西乡峡口至骆家坝等3个项目建成投运，中心城区综合客运枢纽站试运营。水利电力项目方面，汉江综合整治平川段北岸堤防全线贯通，建成330千伏坚强环网并打通第二电源通道。城镇基础设施项目方面，中心城区107个城建重点项目完成投资352.02亿元、占年计划的108.85%，兴汉新区、滨江新区的一批新项目建成投用，打通傥骆路、西环路南段等8条断头路，完成莲湖路等10条城市主干道亮化改造，建成汉宁路西段、南大街南段截污干管，汉宁路地下综合管廊及道路工程加快推进；9个省级重点镇和文化旅游名镇完成投资40.6亿元。

　　（三）精准施策聚合力，三大攻坚战成效明显。脱贫攻坚方面，大力实施脱贫攻坚三年行动，建成农村饮水安全巩固提升工程451处，新改建通村水泥路564公里，略阳县被评为全省“四好农村路”示范县；“十三五”易地扶贫搬迁主体工程提前竣工，危房改造1.6万户；产业扶贫成效明显，带动10.3万贫困户在产业链上增收，城固县产业扶贫经验被省委胡和平书记批示在全省推广；实施苏陕扶贫协作项目269个，落实协作资金2.91亿元，带动贫困人口2.1万人。“互联网+健康扶贫”应用项目被列为国家首批试点，镇巴县荣获全国脱贫攻坚组织创新奖。佛坪县率先脱贫摘帽；全年5.58万户18.47万人脱贫、344个贫困村退出，汉台区整体脱贫，留坝县有望脱贫摘帽。污染防治方面，在全省率先出台生态环境保护岗位责任清单；中央环保督察反馈问题扎实整改，成为督察“回头看”期间全省唯一没有下沉、没有现场督办环境投诉的市；强力开展秦岭生态环境问题专项整治，排查问题475个，完成整改458个、占任务的96.4%；全市空气质量优良天数达331天，成为全省唯一空气质量十项指标全面完成、全面变优的市；河（湖）长制有效落实，272处入河排污口建档立卡、精准管理，市、县污水处理厂提标改造工程全面竣工，中心城区污水直排问题基本解决，主要污染物减排任务超额完成，汉江、嘉陵江出境水质稳定达到Ⅱ类标准，集中式饮用水源地水质达标率100%；完成土地污染状况详查，强化土壤污染风险防控和修复治理，土壤环境质量持续优化；“大棚房”清理整治扎实有效；造林23.91万亩。防范化解重大风险方面，出台和落实加强政府债务管理促进经济发展《实施意见》、政府性债务风险化解《工作方案》，不但实现了有效控增量、减存量、防风险目标，而且通过用足用活政策、有力保障了项目建设和民生事业资金需求；健全政府性融资担保体系，规范市县投融资平台，市投控集团市场化运行模式为全市创出经验，金融生态环境持续优化。

　　（四）补短强弱增后劲，动能活力加速释放。扎实推进供给侧、放管服、投融资等重点改革，园区创新发展、六大产业提质增效等26项具体改革创新措施陆续落地、助力发展；营商环境持续优化，全市行政审批时间缩短30%，优化税收营商环境做法得到刘国中省长肯定，不动产登记等方面经验在全省推广；国企“三供一业”移交全面完成。大力推进创新驱动，培育认定高新技术企业、瞪羚企业61户，1亿元的绿色循环发展科技投资基金正式运营，市科技资源统筹中心建成投用；申报发明专利795件，技术合同交易额2.16亿元；荣获省级科学技术奖10项、居全省第2位；成为全省第三个国家创新型城市试点市。积极探索开放融合新路，京东、启迪控股、碧桂园、恒大等24户世界或国内500强、行业100强企业落户汉中；军民融合、央地融合、部省市融合有力有效，国企进汉、省企入汉成果丰硕，签约项目总投资1210亿元；城固机场联接11个航点，旅客吞吐量49.5万人次、增长19.5%，货邮运输1156吨、增长83.4%。

　　（五）不遗余力惠民生，群众福祉持续增进。全年新增城镇就业6.1万人。有效保障11.68万城乡低保对象、1.98万农村特困人口基本生活，医疗和临时救助25.1万人次，每千名老人拥有养老床位数38张。东辰外国语学校开工建设，北师大汉中学校成功落地，在全省率先启动创建义务教育优质均衡发展县，超大班额治理和普惠性幼儿园建设完成省考指标，高考上线人数实现“七连升”。紧密型医共体建设持续加强，组建全省首家政府主导的健康管理联合体，市级三级医院全面托管留坝、佛坪区域医疗服务。中心城区美丽城市建设三年行动扎实推进，国家森林城市创建通过第三方评估验收，省级环保模范城市创建成功，被新华网评为首批生态型城市；西乡成功创建为国家生态文明建设示范县，留坝、佛坪跻身全省生态建设强县行列；建成省级旅游特色名镇、乡村旅游示范村42个。改造棚户区1.95万户，分配公租房4000套。改造电力“一户一表”4.68万户，一般工商业电价降低10.05%。解决40.66万农村人口的饮水安全问题。累计建成基层综合文化服务中心1858个，免费开放221个“两馆一站”；净增规上文化企业24户、文化产业增加值增长14%。江滨体育公园和市体育运动学校建成投用。农业普查和地名普查工作获全国先进，农民工维权法律援助工作在全国创造经验。成功应对四轮强降雨、勉县黄龙垭滑坡险情和“4?24”粗酚、“6?24”粗苯过境车辆泄露事件，并得到中省肯定。安全生产事故起数、死亡人数继续保持“双下降”。食药监管全面加强，信访维稳扎实有效。扫黑除恶专项斗争深入开展，打掉黑社会性质犯罪组织和涉恶犯罪集团、团伙36个，侦办涉恶类犯罪案件578起，抓获涉案成员838人，全市公众安全感满意率达94.62%、比上年提升0.92个百分点。

　　（六）持之以恒提效能，政府建设不断加强。持续深化“激情、干净、超越”主题作风建设，扎实推进“思想奠基?党性塑造”工程，深入开展“讲政治、敢担当、改作风”等专题教育，严格落实“三项机制”，驰而不息转作风。坚持市政府班子集体学法和重大事项专家咨询制度，出台《汉中市重大行政决策程序规定》，决策机制进一步完善。自觉接受人大、政协监督，全年邀请省、市人大代表、政协委员100余人次列席市政府重要会议，750件人大代表建议、政协提案全部办复。“12345”政府服务热线高效运行，“市长信箱”来信办结率和人民网留言回复率均达100%。从严落实中央八项规定实施细则精神和省委实施意见、市委实施办法，深入整治“慵懒散慢虚”等形式主义、官僚主义问题，持续推进审计监督全覆盖，政府效能进一步提升。在年度社会评价满意度调查中，“两代表一委员”满意度居全省第2位，人民群众满意度、营商环境满意度均居全省第3位。

　　与此同时，人防、档案、慈善、残疾人、地方志、民族宗教、外事侨务、红十字会、民兵预备役等工作，也取得了新成绩。

　　各位代表，过去一年的接力奋斗，汉中圆满收获了改革开放40周年的丰硕成果。40年的砥砺奋进，古老而美丽的天汉大地谱写了思想大解放的凯歌、展现了体制大创新的活力、创造了经济大发展的佳绩、实现了社会大进步的巨变；特别是党的十八大以来，主要经济指标增速高于全国、全省平均水平，汉中的改革开放和现代化事业呈现新气象、站上新起点。这些成绩的取得，是习近平新时代中国特色社会主义思想科学指引的结果，是省委、省政府坚强领导的结果，是市委带领全市人民苦干实干的结果。在此，我谨代表市人民政府，向全市广大干部群众，向驻汉部队官兵，向大力支持政府工作的人大代表、政协委员，向各民主党派、各人民团体和社会各界人士，向在汉中创业的投资者、建设者，表示衷心的感谢并致以崇高的敬意！

　　在肯定成绩的同时，我们清醒地认识到，汉中发展仍面临不少困难和问题：一是产业发展仍有短板，工业上传统产业占比较大，农产品精深加工和高端农产品开发有待加强，全域旅游品牌还不响亮。二是发展动能不够强劲，支撑发展的大项目、产业项目不够多，微观主体活力不够强，融资难、融资贵问题依然突出，营商环境仍需持续改善。三是民生供给质量还不高，教育、医疗、养老等公共服务还不能很好满足群众需要。四是对外开放仍需扩大，特别是与西安、成都、重庆等周边城市的融合发展应持续发力，以增强汉中对优质资源的吸附力。五是作风建设有待深化，一些部门和干部观念落后、作风漂浮、能力不足，工作不扎实、不落实等现象依然存在。我们将坚持问题导向，尽心竭力解决。

　　二、2019年的工作目标、要求和原则

　　今年是新中国成立70周年，是改革开放再出发的开局之年，是推动新时代追赶超越、高质量建设“三市”、全面建成小康社会的关键之年。做好今年政府工作，意义深远、责任重大，必须把握大局、研判形势、主动作为。

　　从当前面临的形势看：一是基础坚实。近年来，我市主要经济指标稳居全省第一方阵，经济发展“稳”的基础不断巩固、“进”的动能持续增强；“六大产业”发展迅猛，多业驱动、多点发力的势头更加强劲；兴汉新区、滨江新区、航空智慧新城等板块强势崛起，城市承载能力不断提升。二是挑战严峻。宏观经济运行稳中有变、变中有忧，经济增长的外部条件进一步趋紧；毗邻地区、省内城市发展竞相赶超，我市“追前甩后”的压力依然很大；站位新时代，化解各类矛盾、防范重大风险、消除短板弱项的任务十分艰巨。三是机遇叠加。中央经济工作会“五坚持、六个稳、两保持、七重点”的决策部署，以及省委、省政府贯彻落实的政策措施，特别是支持民营经济发展、大规模减税降费等政策，将有力支撑我市经济保持中高速增长、实现高质量发展；在当前形势下，发达地区面临经济下行和生态环保的双重压力，而我市多元支撑的产业格局和良好的生态优势，为我们加快转型升级、实现后发赶超提供了时间和机会窗口。只要我们保持战略定力、坚定必胜信心，以新理念、新坐标推动各项工作，就一定能够把握主动、抢占先机、创造未来。

　　今年政府工作的总体要求是：以习近平新时代中国特色社会主义思想为指导，深入贯彻党的十九大和十九届二中、三中全会精神，按照中央经济工作会、省委十三届四次全会和市委五届七次全会的部署安排，统筹推进“五位一体”总体布局，协调推进“四个全面”战略布局，紧扣追赶超越、“五个扎实”要求和“五新”战略任务，突出“绿色循环、生态宜居”战略定位，坚持稳中求进工作总基调，坚持新发展理念，坚持推进高质量发展，坚持以供给侧结构性改革为主线，坚持深化市场化改革、扩大高水平开放，保持高质量发展与中高速增长相统一的发展定力，坚定不移实施“六个三”战略部署、打造“三个六”重点支撑，加快建设现代化经济体系，着力激发微观主体活力，统筹做好稳增长、促改革、调结构、惠民生、防风险各项工作，推动新时代追赶超越、高质量建设“三市”再创新业绩，为全面建成小康社会收官战打下决定性基础，以优异成绩庆祝新中国成立70周年。

　　主要预期目标是：生产总值增长9.2%—9.7%，全社会固定资产投资增长17%左右，规模以上工业增加值增长12%左右，社会消费品零售总额增长12%左右，地方财政收入同口径增长6%，城乡常住居民人均可支配收入分别增长8.5%、9.2%左右。特别要全力确保实现“七个翻番”，即：在2012年基础上，生产总值、人均生产总值、规上工业增加值、服务业增加值、人民币存款余额翻一番，旅游总收入翻两番，全社会固定资产投资增长三倍。以上指标设置，体现了主动应对宏观经济变化的坚定信心，体现了汉中发展的阶段性特征，体现了“跳起来摘桃子”的加压奋进精神，这是汉中新时代追赶超越、高质量建设“三市”的必然要求和坚定不移的目标。

　　实现上述目标，我们将遵循以下工作原则：

　　第一，聚焦“三市”建设目标，完善规划、汇聚要素，全面构建发展格局。全力打造支撑平台。围绕做强“三个六”支撑，发挥规划的拉动作用，高点起步、有的放矢，做实做精各类规划；发挥好龙头企业引领作用、生产性服务业配套作用和骨干产业园区承载作用，优化产业布局和要素配置，不断延链补链强链，促进“六大产业”集群集约发展；发挥城镇的承载和聚集作用，打造“四化”协同互动新格局。增强微观主体活力。建立健全现代市场体系，推动资源要素市场化配置，以小政府撬动大市场，以宏观创新激活微观主体；高度重视非公有制经济发展，激发民营经济创造活力。加快补齐要素短板。树立战略眼光，紧盯科技创新和产业发展前沿，坚持不求所在、不求所有、只求为我所用，加大科技团队引进、领军企业扶持和种子企业培育，以产业融合为抓手，推动新兴产业蓬勃发展。

　　第二，坚持以人民为中心，生态优先、绿色发展，走好“三生融合”道路。加大“融”的力度。坚持从生态文明建设的高度深化认识，发挥生态资源优势，优化生产空间布局，彰显生活美好图景，实现从兼顾平衡到互动协同、再到相互渗透深度融合的飞跃。把握“融”的关键。用好市场机制，激发市场活力，把“三生融合”与一二三产融合、城乡发展融合和军民、央地、部省市融合协同推进，实现高层次、多维度融合发展。提升“融”的水平。出台“三生融合”发展实施意见，建立健全配套的政策、标准、法规、责任体系，不断优化生产、生活、生态空间布局。

　　第三，立足区位人文特点，内外兼修、扬长补短，着力提升城市能级。提升内在品位、增强亲和力。全力推进美丽城市建设，不断提升幸福指数和城市“温度”；全面优化服务环境，切实提高群众满意度；坚持以文铸魂、以文化人、以文塑城，着力涵养城市精神。扩大对外辐射、提升影响力。按照市委总体部署，逐一制定专项规划，全力打造“六个中心”，加快建设区域中心城市，不断扩大对外辐射、引入外部资源，使汉中成为亲商聚商稳商富商的兴业热土。内外融合互动、创造新实力。出台具体政策措施，积极融入丝绸之路经济带、长江经济带和京津冀经济圈，特别是西安、成都、重庆等周边城市，促进高端要素与汉中资源深度融合，实现资源共享、优势互补。

　　第四，正视各种短板弱项，深化改革、扩大开放，充分释放潜能活力。改革攻坚破瓶颈。聚焦“三大变革”，下大力气破除体制机制弊端，使市场在资源配置中起决定性作用，更好发挥政府作用，着力解决要素聚集、融合发展、产业转型、区域协同等方面难题，力促经济社会发展稳得住、进得快。开放聚力强动能。根据市委确立的框架思路，坚持“一个中心一个工作专班一套推进方案”，加快启动建设“六个生产交易中心”，做强主体业态，支撑“三个经济”发展，着力构建开放型经济体系。创新引领赢主动。紧扣矛盾新变化和战略新机遇，更多运用市场化、法治化手段，大胆探索、先行先试，变压力为动力、化风险为机遇，为高质量发展注入强劲动能。

　　三、今年重点工作任务

　　做好今年工作，我们必须以终为始、强力突破，加速推动新时代追赶超越、高质量建设“三市”。重点做好以下八个方面工作：

　　（一）坚决打好三大攻坚战，为新时代追赶超越高质量建设“三市”增添后发优势。突出靶向施策、精准发力，高质高效完成年度目标任务。

　　打好精准脱贫攻坚战。聚焦“两不愁三保障”，把脱贫攻坚与乡村振兴相结合，坚持目标标准，贯彻精准方略，确保剩余8个贫困县区摘帽、518个贫困村退出、11.8万贫困人口脱贫。突出抓好产业扶贫，扎实推进“七大行动”，大力发展“5+X”特色产业，健全带贫益贫长效机制，让贫困群众在产业链上稳定脱贫。深入推进基础设施和“两房”建设，9月底前，所有贫困村的安全饮水、村组道路、农网改造、网络覆盖、标准化卫生室全面建成达标。统筹抓好行业扶贫，推进健康扶贫“汉中模式”再升级；资助1.7万名贫困家庭学生；续聘5000名生态护林员；保障好7.6万特殊困难群众基本生活。深化通汉扶贫协作和社会帮扶，加速推进227个协作项目，落实国企合力团10个帮扶项目，组织600家民营企业、300家社会组织参与扶贫。落实好“1+10”配套政策，市财政安排1000万元以上的深度贫困县专项扶贫资金，每个深度贫困村整合投入涉农资金不低于500万元，全力支持略阳、镇巴两个深度贫困县和110个深度贫困村脱贫退出。持续推进志智双扶，增强贫困群众自我发展能力。扎实做好中央脱贫攻坚专项巡视反馈问题整改。巩固提升脱贫成效，妥善解决收入水平略高于建档立卡贫困户的群众缺乏政策支持等新问题，防止贫困人口返贫。

　　打好污染防治攻坚战。深入推进“四个保卫战”，出台秦巴生态保护治理实施意见，全面推行“山长制”，持续深化秦岭生态环境专项整治，严厉查处违法搭建、排放、采挖、砍伐等行为；启动平川县大气和水质动态监控项目，坚持减煤、控车、抑尘、治源、禁燃、增绿六措并举，消除常规气象条件下的重度污染天气，确保中心城区空气优良天数不低于290天；严格落实河（湖）长制，扎实开展最美河湖修复行动，抓好汉江、嘉陵江流域环境突出问题专项整治，加强水源地保护，确保全域水质保持优良；建立污染地块名录和负面清单，抓好重金属、固体废物污染等综合治理，确保土壤污染“零增量”“减存量”；3月10日前全面完成“大棚房”清理整治。系统治理生态，充分发挥生态修复公司作用，抓好矿山地质环境恢复治理、尾矿库和矿山弃渣综合利用、河湖清淤疏浚等工作；积极开展国土增绿行动，持续推进天然林保护、退耕还林、水保治理和重点区域绿化；深化“三保三治”，加强地质灾害监测防治；实施农村土地综合整治，设立耕地保护基金，严守耕地保护红线和城镇开发边界。出台生态文明建设指导意见，推动环境保护从被动监管向主动优化转变。全力推进汉江生态经济带建设，着力打造汉江干流百公里生态景观和生态经济廊道。

　　打好防范化解重大风险攻坚战。防控政府债务风险方面，加强政府债务管理，规范政府购买服务，建立政府举债负面清单制度，实行债务问题倒查和终身问责；推动政府举债行为向市场化融资转变，用足用好政策，以市场化手段谋划和运作项目，善发力、巧用力，堵后门、开前门，坚决遏制增量、妥善化解存量。防控金融风险方面，规范政府性融资平台和政府投融资行为，深化金融市场清理整顿，建立非法金融监测预警平台，坚决打击非法集资、金融诈骗、恶意逃废债务等行为。防控房地产领域风险方面，坚持“房子是用来住的、不是用来炒的”定位，完善土地、税收、金融、住房公积金等政策，健全住房市场和住房保障体系，合理引导市场预期和购房行为，促进房地产市场平稳健康发展。

　　（二）聚力推进项目建设，为新时代追赶超越高质量建设“三市”打造“四梁八柱”。根据区域特点，依托资源禀赋，深挖潜力、整合资源，做大存量、引入增量，力促项目大突破。全年实施重点项目403个，年度投资811.4亿元。

　　高起点策划包装。围绕“三大项目支撑体系”，聚焦“六个中心”“六个生产交易中心”“六大产业集群”建设，坚持规划拉动，引进专业团队，用大产业、大园区、大基地理念，重点在高端装备制造、新兴产业发展、新型能源利用、生态旅游开发和基础设施提升等方面，精心策划一批大项目、好项目，特别是投资百亿元以上的支柱型项目和延链补链强链的产业类项目，为长远发展提供有力支撑。

　　大力度推进建设。严格落实项目动态管理和“五个一”工作机制，坚持领导包抓促动、集中开工牵动、示范引领带动、考核倒逼推动，力促陕西首铝铝合金模板系统、勉县超级电容电极材料等64个收尾项目全面竣工，神龙药业功能食品、圣水大鲵产业示范园、西镇高速、镇巴星子山隧道及引线工程、引汉济渭等150个续建项目加快推进，陕飞公司特种飞机生产线、启迪科技城、汉中机场改扩建、青海至河南800千伏直流输电工程汉中段等97个新开工项目尽早启动，天坑开发、天和防务、君毅航空产业园、燃气热电联产等92个重大前期项目取得突破。

　　全链条服务保障。着眼项目建设全过程，树牢服务理念，优化服务环境，提供“一条龙”“保姆式”服务。深化政银企合作，发挥投融资平台作用，激活民间资本；积极争取专项债券，规范PPP项目运作，拓宽项目融资渠道。健全土地大收储制度，用好占补平衡、增减挂钩政策，提高土地集约利用水平，全力保障项目用地。严厉打击恶意阻工、强揽工程等行为，净化项目建设环境。

　　多渠道招大引强。坚持高水平规划引领，聚焦国际国内500强、行业100强等重点企业，瞄准京津冀、长三角、港澳台及关天、成渝、江汉经济圈等重点区域，用好“汉商”回归政策，做好第四届丝博会主题市参展工作，办好陕南绿色循环合作交流活动，借助陕粤港澳经济合作周等平台，强化精准招商、专业招商、产业链招商，着力引进一批大项目、大企业、大集团。特别要突出产业生态的建立完善，重视引进融合特点明显的新业态项目；跟踪衔接益海嘉里、启迪控股、惠威科技、华强方特等重点企业，力促合作项目取得突破。全年引资到位808亿元、增长10%。

　　（三）提质增效壮大工业，为新时代追赶超越高质量建设“三市”做强骨干支撑。聚焦新旧动能转换，完善产业集群发展规划，积极构建现代工业体系，推动实体经济高质量发展。全年实施重点工业技改项目50个，实现规上工业总产值1765亿元、增长12%左右。

　　加快转型升级。大力实施“产业+”战略，推进中国制造2025汉中实施方案，加快支柱产业向高端化、绿色化、集群化迈进。装备制造方面，强力推进民用货机、运X飞机生产及总装扩能配套、新能源汽车及零部件等项目，壮大提升传统优势产业。现代材料方面，积极推进产品换代、生产换线，加快推进汉钢短流程炼钢工艺改造、有色全产业链材料园、汉圆装配式建筑等项目，促进钢铁转型、有色做强。高品质食药方面，以产业链规划为引领，发展有机产业和中医药产业，振兴“白、黄、黑”酒业，做大“公主”品牌，加快天谷药业中药材加工及物流、汉江药业高端原料药新区、通城食药产业园等项目建设，提升食药产业竞争力。新兴产业方面，深入实施“5353”培育工程，全力推进京东电子商务运营及物流结算中心、中航电测智能应变式传感器与核心芯片智能制造、移动ICT数据中心等项目，促进战略性新兴产业加速成长。新型能源方面，推进镇巴天然气、南郑页岩气勘探开发，加快LNG分布式能源、宁强至汉中天然气输气管道、中环垃圾焚烧发电等项目建设，推动氢能、生物质能等新能源开发利用。

　　做优产业链条。聚焦六大产业，突出市场导向，强化项目支撑，积极培育行业龙头企业、节点支撑企业、上下游配套企业，健全技术创新、供需链接、要素保障、组织服务等延链补链机制，全力打造航空装备、汽车制造、工具仪表、新型建材、有机食品、中药材等若干高端产业链，加快形成产业集群。启动实施现代服务业创新发展三年行动计划，放宽市场准入、完善行业标准、创造良好条件，大力发展现代物流、研发设计、融资租赁、信息服务等生产性服务业，为企业提供全方位、全生命周期服务。

　　强化平台支撑。全面落实工业稳增长措施，加强重点企业运行协调和跟踪监测，用好工业发展专项基金，撬动社会、产业和金融资本向实体经济聚集，推动骨干企业扩投资、稳增长。统筹推进产业链、创新链、资金链、政策链有机融合，加快建设4个千亿级骨干园区和8个百亿级县级园区，做强产业平台支撑。出台支持民营经济高质量发展的若干意见，设立5亿元的民营企业纾困基金，降低融资、用地、物流等成本，促进民营企业做大做强。搞好第四次经济普查。实施扶助中小微企业专项行动，全年新培育“四上”企业360户、市场主体2.8万户，形成经济增长新动能。

　　（四）大力实施乡村振兴，为新时代追赶超越高质量建设“三市”构筑坚实基底。认真贯彻中省农村工作会议精神，深入实施乡村振兴战略，在实现农业农村现代化征程上迈出新步伐。

　　提升现代农业水平。深化农业供给侧改革，推进十大绿色农产品基地提档升级，确保粮油、茶叶、水果、蔬菜产量分别达到117万吨、4.25万吨、50万吨、250万吨。积极发展生态农业、休闲农业、创意农业等新业态，支持大鲵、乌鸡等特色养殖，抓好田园综合体开发建设，着力打造现代农业园区。加快推动特色农产品精深加工和高端农产品开发，促进一二三产融合发展。强化“三品一标”认证管理，用好资本和市场的力量，有效整合特色品牌，让“汉字号”农产品畅销国内外。毫不松懈抓好非洲猪瘟等重大动物疫病防控工作。

　　深化小农户对接大市场。创新利益联结机制，积极培育新型经营主体，支持农业龙头企业做大做强，力争重点农业企业销售收入突破140亿元；依托京东云仓、社员网、农村淘宝等平台，深化农村电商全覆盖；高质量办好第四届中国有机农业大会，大力发展订单农业和智慧农业，继续在一线城市设立汉中名优产品专营店、展销厅，举办系列推介促销活动，加快完善产加销链条，推动农业增效、农民增收。

　　建设美丽宜居乡村。突出规划引领、示范带动，打造20个乡村振兴示范村。坚持“一村一方案”，严格村庄规划建设许可，推进乡村综合整治。以建设“四好农村路”为重点，提升农村基础设施和公共服务水平。深入开展农村人居环境整治三年行动，重点推进农业面源污染治理、垃圾污水处理、厕所革命和村容村貌提升，抓好10条美丽乡村风景线和市级示范村建设，共建生态宜居的美丽家园。振兴乡村文化，弘扬文明乡风、良好家风、淳朴民风，健全自治、法治、德治相结合的乡村治理体系。

　　稳步推进农村改革。深化农村土地制度改革，探索多种形式的“三权分置”模式，力争适度规模经营面积占比达到28%以上。积极推进农村集体产权制度改革，盘活资源资产，壮大集体经济。深化农业经营体系改革，大力发展参股经营、委托经营等多种联合开发模式。充分发挥振兴乡村产业服务公司在引导工商资本参与乡村振兴中的作用，推动土地、资金、产业等要素向农村汇聚。加强乡土人才培养，鼓励支持各类人才领办创办经济实体。支持农业保险发展。帮助农民就地就近创业就业，多渠道增加农民收入。

　　（五）深度融合文化旅游，为新时代追赶超越高质量建设“三市”培育强劲引擎。围绕建设文化旅游强市，全力推动文化旅游深度融合，促进三产消费持续繁荣。

　　全域化推进。坚持市县（区）联动、点线面结合，抓好示范县创建，确保全域旅游示范市验收顺利通过。加快紫柏山、华阳、黎坪、青木川创5A步伐和南湖、龙头山、汉水源等景区综合开发，抓好天汉文化公园、龙岗文化生态旅游园区、西汉三遗址、国际研学小镇、张骞文化旅游园区、“秦岭四宝”旅游区、九昱汉水温泉等项目建设，大力发展特色乡村旅游，串珠式打造一批精品景点和精品线路。创新举办油菜花节、朱鹮国际论坛、丝路国际旅游合作论坛等品牌活动，强化宣传营销，提升汉中全域旅游美誉度。全年接待游客突破6000万人次、旅游总收入超过350亿元，分别增长15%以上。

　　全业态融合。大力实施“旅游+”战略，积极吸纳西安、成都、重庆等区域特色旅游要素进入汉中，加快打造“汉家乐”品牌，大力发展生态旅游、研学旅游、康体养生等新业态，推动旅游与文化、生态、农业、体育等深度融合，全业态延伸旅游产业链条，构建大旅游、大市场、大产业格局，引领三产繁荣发展。

　　全方位服务。树立以游客为中心理念，抓好重点景区旅游专线、游客集散中心、智慧旅游平台、旅游厕所等设施建设，打造多式联运、畅通高效、安全舒心的服务体系；积极开发特色旅游商品，严厉整治旅游环境，强化住宿、餐饮、出租车等行业规范化管理，提升旅游服务的人性化、精细化、标准化水平。

　　全过程引领。深入推进“五个一”精品工程，完善公共文化服务体系和文化设施，抓好市县融媒体中心建设，启动汉中广播电视台广电中心二期工程，广泛开展群众文化活动，推出《凿空者》《红色交通线》等一批文艺精品力作。深度挖掘地域文化，加强文物、文化遗产保护利用，推进藏羌彝文化产业走廊、天汉文化广场等项目建设。大力实施“文化+”行动，用好文化旅游产业发展基金，培育壮大文化企业和新兴文化业态。坚持以文兴旅，借助“兴汉胜境”等平台，办好世界汉文化、汉学、汉语三场大会，不断扩大“汉文化”影响力。

　　全维度带动。加快提升现代服务业，推动传统商贸线上线下融合，大力发展假日、会展、数字、共享经济，办好“中国汉中首届美食文化节暨地方土特产品展销会”“汉中O2O国际汽车博览会”等活动，培育汽车、养生养老、文化教育等消费热点，着力增强消费拉动力，全年社会消费品零售总额达到526亿元。

　　（六）开放改革创新协同，为新时代追赶超越高质量建设“三市”激发蓬勃活力。以改革开放再出发为契机，以更大的魄力、更有力的举措，完善体制机制，激发动能活力。

　　推动开放融合。大力发展“三个经济”，加快汉中机场改扩建和“155”产业集群公路建设，构建陆空互动、多式联运的立体交通网络；加强与京东、浪潮集团合作，推进信息化综合服务平台和区域云计算大数据产业基地建设；高标准建设褒河物流园，梯次打造商贸、冷链、口岸国际等“五大物流园区”，加快汉中海关通关运行，促进发展要素在汉中汇聚融合、高效流动。全力引进大型企业集团在汉中设立分部机构，抓好苏陕产业“园中园”、西安“汉中飞地”及离岸孵化基地等平台建设，吸纳高端资源进入汉中。积极推动军民、央地和部省市融合，促进国企资金、技术、管理、品牌优势与汉中产业深度融合。支持装备制造、机械电子、特色农产品等优势产业抱团出海，不断提升经济外向度。

　　深化重点改革。持续推进供给侧改革，推动落后产能、过剩产能和“僵尸企业”出清；落实好减税降费政策，切实降低企业成本。深化放管服改革，坚持标本兼治，扎实开展优化提升营商环境十大专项行动和三年计划，借助社会力量倒逼推进；组建市、县（区）行政审批服务局，推进相对集中行政许可权改革市县全覆盖和“证照分离”改革；深化“互联网+政务服务”，推行“一网通办”等经验。深入推进投融资改革，出台投融资改革实施意见，健全市、县（区）政府性融资平台，加快融资平台市场化转型，建成市综合金融服务平台；探索“财政+投融资平台+金融资本”等运作模式，盘活存量资金，撬动社会资本；积极争取各类金融机构在汉设立分支机构，扩大与各类基金及上市公司的合作，支持优质企业上市融资、发行债券；推进信用信息共享，建立降低中小微企业融资成本的奖补机制和信用背书机制，有效缓解融资难、融资贵问题。积极推进要素市场化改革，深化资源类产品和公共服务价格改革，出台科技、金融等资源要素汇聚的措施办法，助力现代产业发展。加快国企国资改革，完善“三重一大”决策机制，健全现代企业制度。稳步推进市县政府机构改革，深化事业单位分类改革，优化行政管理体制和政府职能。

　　坚持创新驱动。以国家创新型城市建设为抓手，完善以企业为主体、市场为导向、政用产学研相结合的协同创新体系。加快建设启迪汉中氢能与燃料电池工程技术中心，发挥西工大军民融合研究院、陕西理工大学、市绿色循环暨产业经济研究院和科技资源统筹中心等机构的作用，引入六大类产业研究机构，加大科研经费投入，在装备制造、人工智能、生物医药等方面，集中攻克关键核心技术，推动科技成果转化运用，提升产业创新力和竞争力。新培育高新技术企业、瞪羚企业、科技型中小企业100户；新建企业孵化器、众创空间和科技服务平台12个。

　　（七）全面提升城市品位，为新时代追赶超越高质量建设“三市”开拓广阔空间。坚持产城人景业深度融合，推进陕西最美城市建设，不断提升城市品位和能级。

　　完善基础设施。全力推进中心城区美丽城市建设三年行动和“五个十大”重点工程，全年计划实施城建重点项目74个、投资230亿元。加快天汉楼、大剧院、经创中心、信息大厦、地下综合管廊、棚户区改造、重点镇“两厂（场）”、长林水源地等项目建设，启动实施中心城区雨污分流四年整治计划，着力补齐城市发展短板。巩固国卫、国园等创建成果，建成国家森林城市，加快国家文明城市创建步伐。

　　推进精细管理。聚焦群众衣食住行日常需要，关注小事、注重细节、讲求实效，完善停车场、充电桩、公厕、人行天桥等便民服务设施，规划建设一批特色美食街、精品商业街、历史文化街，推进绿化亮化、街区改造、环境整治，加强人性化、精细化、规范化管理，打造高效、便捷、规范的生活服务圈，让人们随处可以感受到灵动的人文气韵和浓厚的生活气息，让城市生活更便利、更舒心、更幸福。

　　统筹区域发展。加快汉台、南郑一体发展、提升能级，推动城固、航空智慧新城产城融合、良性互动，支持洋县、西乡、宁强、镇巴循环发展、绿色强县，力促勉县、略阳转型升级、挖潜增效，鼓励留坝、佛坪一业突破、振兴乡村，协同抓好省市重点镇、文化旅游名镇、特色小镇建设，实现错位发展、板块联动、城乡融合。

　　讲好汉中故事。坚持高水平策划宣传，深度挖掘“昨天”的历史文化精粹，全面总结“今天”的改革发展成就，充分展示“明天”的美好愿景，让全国乃至世界深入了解汉中。积极培育和践行社会主义核心价值观，深入推进公民思想道德建设，广泛开展群众性精神文明创建活动，着力提升市民文明素质。全面完成第二轮志书编纂任务。深入提炼汉中城市精神，激发正能量、展示精气神，将鲜明的城市形象立起来、推出去。

　　全力聚集人气。制定优惠政策和具体办法，叫响做靓“四个在汉中”城市品牌。聚焦“学研在汉中”，加强与中科创大、北京启迪等文化教育集团合作，全力引进优质教育资源兴办品牌教育，加快建设东辰外国语学校和北师大汉中学校；支持陕西理工大学建设，引入知名大学在汉设立分校；统筹推进公办学校教育资源优化配置，不断提高办学质量；实施名院名所引进工程，吸引高校、院所、企业资源，创建一批应用型研发机构，加快国际学生感知汉文化基地建设，积极举办国内外知名学术论坛，让汉中成为优教研学的首选之地。聚焦“医养在汉中”，多渠道引进外埠资源，创办一批高端医养机构，大力发展康体健身、医养结合等产业，引导和激励社会资本进入养老产业，建成一批示范项目，把汉中打造成“绿色康养胜地”。聚焦“旅居在汉中”，积极发展“酒店式公寓”“产权式酒店”，实现生活服务与异地对接，推动旅游向旅居转变，让更多人留在汉中、带来资源。聚焦“兴业在汉中”，坚持以优美愿景感召人、优惠政策吸引人、优厚待遇留住人、优质环境待好人，拿出最优质的资源吸纳高端人才和客商汇聚汉中；推行“孵化+创投”等模式，搭建创业创新平台，让“打工一族”早日回归，让更多人在汉中放心创业。

　　（八）全力以赴改善民生，为新时代追赶超越高质量建设“三市”凝聚强大合力。持续保障和改善民生，让群众有更多、更直接、更实在的获得感、幸福感、安全感。

　　健全完善社保体系。实施更加积极的就业政策，开展稳就业精准帮扶行动，统筹抓好高校毕业生、退役军人、返乡农民工、就业困难人员等重点群体就业，全年新增城镇就业3.3万人、转移农村劳动力70万人。健全企业职工养老省级统筹制度，稳步提高退休人员基本养老金。全面落实低保提标和高龄老人补贴、残疾人优待等政策；加大社会救助力度，有力保障困难群众基本生活。做好农村“三留守”人员、城镇弱势群体关爱服务。以居家为基础、社区为依托、机构为补充、医养相结合，加快构建多层次养老服务体系。新建农村社区服务中心36个、省级标准化社区8个，提升改造27个城乡社区基础设施。大力发展公益事业。认真落实优抚安置和保障待遇政策，切实维护退役军人合法权益。

　　优先发展教育事业。高度重视教育资源配置不均衡和农村学校师资水平下降、学生流出严重等公办教育短板问题，加大教育资源优化配置力度，扩大普惠性学前教育资源，加快农村寄宿学校、小规模学校和中心城区学校建设；推行义务教育免试就近入学，着力解决“大班额”、“择校热”、中小学生课外负担重等问题；重视青少年心理健康教育，促进义务教育阶段学生体质健康达标。积极开展省级标准化高中创建，加快普及高中阶段教育。抓好质量提升“316”工程教育督导评估，创建10所省、市教育质量提升优质学校。创办特色职业教育，规范社会力量办学，完善终身教育体系。深化教师队伍改革，加强师德师风建设，落实好乡村教师生活补助等政策。

　　加快健康汉中建设。树立“大卫生、大健康”理念，编制完成《健康汉中2030规划纲要》，推进健康城市、健康县城和8类“健康细胞”示范建设，提升城乡居民健康素质。持续深化以支付方式为抓手的医疗医保联动改革，全力推进紧密型医共体建设，抓好“互联网+医疗”试点，完善120急救服务体系，健全分级诊疗、药品供应等制度。深入实施基层医疗卫生服务能力提升三年计划，健全医疗服务对口帮扶体系，强化疾病预防控制和公共卫生服务。推进中医药事业传承发展。严格落实公共场所禁烟措施。加快十四届全运会汉中场馆建设，建成170个村级农民体育健身工程，办好全省第二届全民健身运动会和“一带一路”国际摩托艇公开赛，精心组织开展群众足球及校园足球系列联赛。

　　推动社会治理创新。扎实开展“安全生产措施落实年”活动，推进双重预防机制，落实“一企一策”措施，加强非煤矿山、道路交通、危化品等重点领域的专项整治，有效遏制较大事故，坚决杜绝重特大事故。强化食品安全源头治理和药品安全监管，每个县区打造5至10个食品安全示范街区或示范商圈，全力创建国家食品安全示范城市。学习推广“枫桥经验”，推进城乡社区治理和服务创新，充分发挥群团桥梁纽带作用，深化“七五”普法和“法治汉中”建设。加大矛盾纠纷排查化解力度，抓好积案化解和重要节点信访工作，有效控制进京非访和赴省集体访。加快“智慧城市”和“雪亮工程”建设，完善治安防控体系；深入开展以扫黑除恶专项斗争为重点的严打整治行动，严厉打击“盗抢骗”“黄赌毒”等犯罪。统筹做好应急管理、防灾减灾救灾等工作。

　　各位代表，在全力做好以上八个方面工作的同时，我在此代表市政府向全市人民郑重承诺，今年突出办好两个方面的大事和实事。一是建成“十大工程”，向新中国成立70周年献礼。成立工作专班，强力推动落实，确保全市脱贫摘帽、汉江干流综合治理全线竣工、阳安铁路扩能改造竣工通车、高铁站南北广场及综合交通枢纽投入使用、兴汉新城文化旅游景区开园、天汉文化广场天汉楼落成开放、陕飞运X飞机系列项目投运、汉钢短流程炼钢工艺改造工程投用、褒河物流园扶贫开发绿色农产品展示交易中心等建成、中心城区污水处理厂等一批城市基础设施竣工。二是办好“十件民生实事”，更好顺应人民美好生活向往。建成农村公路350公里，实现全市行政村100%通沥青（水泥）路；实施农村饮水安全巩固提升工程280处，农村自来水普及率达90%以上；9月底前，“十三五”期间确定的14.7万户易地扶贫搬迁和今年2317户危房改造任务全部完成；加大中心城区“断头路”改造力度，在抓好汉宁路、金华路等14个在建项目的同时，再启动实施至少10个项目；新（改扩）建中小学和幼儿园100所；延伸“互联网+健康扶贫”创新应用，各县区建成移动家庭医生签约服务系统；政府购买公共文化演出1400场次，演出覆盖60%以上的行政村；创建100个就业示范基地和100个社区工厂，让10万名贫困劳动力稳定就业；扶持发展民办养老机构6个，新建日间照料中心20个、农村互助幸福院100个，每千名老人养老床位达到39张；市县政务服务事项网上可办率达到70%以上。

　　四、建设人民更加满意的服务型政府

　　为人民服务、对人民负责、受人民监督、让人民满意，是新时代政府工作的根本取向。我们必须顺应时代要求和人民期待，全面建设人民更加满意的服务型政府，为新时代追赶超越、高质量建设“三市”提供有力保证。

　　（一）坚定政治立场，坚决做到守规矩葆忠诚。旗帜鲜明讲政治是服务型政府的首要特性。我们将深入学习贯彻习近平新时代中国特色社会主义思想，牢固树立“四个意识”，切实增强“四个自信”，坚决做到“两个维护”；扎实开展“不忘初心、牢记使命”专题教育，锤炼“五个过硬”、增强“八种本领”，筑牢信仰根基、增强守正能量；始终恪守政治责任、严守政治纪律，深刻汲取秦岭北麓西安境内违建别墅问题教训，不折不扣贯彻执行中央各项决策，全力落实省委、省政府和市委各项部署，确保政令畅通、令行禁止。

　　（二）深化依法行政，坚决做到明法纪守红线。规范高效讲法治是服务型政府的基本遵循。我们将牢固树立法治思维，大力弘扬宪法精神，严格依照宪法法律行使职权；自觉接受人大依法监督和政协民主监督，高质量办理人大代表建议和政协提案，坚持邀请人大代表、政协委员列席政府重要会议，严格执行重大行政决策法定程序，提升决策科学化、民主化水平；深化行政执法体制改革，严格公正文明执法，对环境保护、食药安全、市场监管等重点领域加大执法力度，有效保障群众切身利益。

　　（三）勤勉履职尽责，坚决做到敢担当善作为。为民务实讲担当是服务型政府的核心要求。我们将强化为民造福、谋求复兴的政治担当，坚持以人民为中心，着力解决群众最关心最直接最现实的利益问题，带领全市人民逐梦新时代、共创新生活；强化勇立潮头、奋勇搏击的使命担当，不畏风险、知难而进，敢为人先、大胆创新，坚定不移推动发展大跨越；强化守土负责、守土尽责的责任担当，不驰于空想、不骛于虚声，主动作为、激情干事，一件一件抓落实，一张蓝图绘到底。

　　（四）从严正风肃纪，坚决做到知敬畏严要求。清廉守纪讲正气是服务型政府的底线标尺。我们将以政治建设为统领，严格履行主体责任和“一岗双责”，始终把纪律规矩挺在前面；以作风建设为抓手，严格落实中央八项规定实施细则精神，持续深化“激情、干净、超越”主题作风建设，严厉整肃不敬畏、不在乎、喊口号、装样子等问题，大力压减规范督查检查考核事项，坚决防止“四风”反弹回潮；以制度建设为根本，推进财政预决算公开和绩效管理，加强审计监督，严格控制“三公”经费，压减5%的一般性支出，把更多财力用于民生改善；以纪律建设为保障，持续加大反腐力度，巩固发展反腐败斗争压倒性胜利，一体推进不敢腐、不能腐、不想腐，着力营造海晏河清的新气象。

　　各位代表，携手新时代，我们要强化奋斗者的使命担当；迈上新征程，我们要焕发追梦人的激情力量。让我们高举习近平新时代中国特色社会主义思想伟大旗帜，在市委领导下，凝心聚力、接续奋斗，再创汉中新时代追赶超越高质量建设“三市”新辉煌，以优异成绩庆祝中华人民共和国成立70周年！
